# A rare case of hypertensive urgency caused by linezolid was reported: A case report

**DOI:** 10.1097/MD.0000000000036328

**Published:** 2023-12-01

**Authors:** Jingjing Luo, Xinan Wu, Yiming Liu

**Affiliations:** a Department of Clinical Pharmacy, Hefei BOE Hospital, Hefei, P.R. China; b Department of Orthopaedics, Hefei BOE Hospital, Hefei, P.R. China.

**Keywords:** blood pressure, case report, hypertension, hypertensive urgency, linezolid

## Abstract

**Rationale:**

Linezolid itself is rarely reported to cause blood pressure elevation, and it is rare to report that linezolid causes hypertensive urgency.

**Patients concerns:**

This case report describes a 38-year-old man who developed acute hypertension after a postoperative foot infection that was treated with linezolid antitherapy. Hypertensive urgency occurred without obvious potential interaction between linezolid and drugs. After receiving appropriate treatment and stopping medication, the patient’s blood pressure returned to normal and did not recur.

**Diagnoses:**

Hypertensive crises occurred during the treatment of linezolid.

**Interventions:**

After stopping linezolid, the patient’s blood pressure gradually returned to normal.

**Outcomes:**

The patient’s blood pressure returned to normal on the 26th day after stopping linezolid, and no abnormal blood pressure was found in the follow-up 2 months after discharge.

**Lessons:**

Linezolid is rarely reported to cause elevated blood pressure, even though it may occur in the absence of obvious drug interactions. Case reported fewer reasons may be for clinicians statistically insignificant or notice, and hypertensive urgency often lead to clinical risk, should be given enough attention to clinical. Pay attention to blood pressure monitoring during use, when there is abnormal increase in blood pressure, should consider adverse drug reactions, give timely discontinuation and give symptomatic treatment.

## 1. Introduction

Linezo1id, the chemical name is (S)–N-[[-3-(3-Fluoro-4-(4-morpho-lino) phenyl)-2-oxo-5-oxazolidinyl] methyl]-acetamide, getting listed on the American FDA approval in 2000, is the first chemical total synthesis of a new type of well applied to clinical azole alkane ketone antimicrobials, and mainly used to treat infections caused by methicillin-resistant *Staphylococcus aureus* or vancomycin-resistant enterococcus.^[1]^ Linezolid has been approved for the treatment of infections caused by vancomycin-resistant Enterococcus faecium, hospital-acquired pneumonia caused by *S aureus*, complicated skin and skin structure infections, uncomplicated skin and skin structure infections caused by methicillin-susceptible *S aureus* or Streptococcus pyogenes, and community-acquired pneumonia caused by Streptococcus pneumoniae.^[1]^ In recent years, linezolid has been widely used in clinical practice. This could increase the risk of adverse drug reactions, notably hematological disorders (anemia and thrombocytopenia), neuropathy, or lactic acidosis have been reported more frequently.^[2,3]^ The adverse reaction of Hypertensive urgency caused by linezolid is very rare.

## 2. Case presentation

The patient, a 38-year-old male, was admitted to hospital on July 19, 2022 due to “bleeding and pain in the right foot for 5 hours caused by heavy object injury.” Physical examination on admission: body temperature 36.6°C, heart rate 78 times/min, respiration 19 times/min, blood pressure 125/75 mm Hg (1 mm Hg = 0.133 kPa). Admission diagnosis: metatarsal fracture (right foot 1–5 metatarsal fracture), open foot injury (right foot open trauma), foot injury (right foot crush injury). 7.19 Emergency debridement and suturing of the right foot was performed. 7.23 Postoperative wound infection accompanied by fever was performed on the patient, and the highest temperature was 38.7°C. 7.25 The wound secretion culture result was Staphylococcus epidermidis, multi-resistant, and vancomycin (1.0 g, ivgtt, q8h) was given. 7.27 The patient’s temperature returned to normal. 8.3 The blood concentration of vancomycin was measured at 6.55 µg/mL. 8.5 Debridement + necrotic tissue removal + metatarsal fracture reduction and internal fixation + wound closed negative pressure drainage were performed. The patient had fever for 5 days after surgery, with a maximum temperature of 38.9 °C. 8.10 Changed to linezolid (0.6 g, ivgtt, q12h), 8.11 The patient’s temperature returned to normal. Debridement and free flap transfer were performed on August 16 and August 22, respectively. 8.31 Stop linezolid. On September 18, he was discharged from hospital. Other main treatment drugs during admission include: Ketorolac tromethamine Injection, Acetaminophen oxycodone, Nadroparin calcium, etc.

On October 6, 2022, the patient was readmitted to hospital due to “redness, swelling and fluid seepage after right foot trauma for more than half a month.” Physical examination on admission: physical examination on admission: body temperature 36.5°C, heart rate 78 times/min, breathing 20 times/min, blood pressure 127/78 mm Hg (1 mm Hg = 0.133 kPa). A 2 × 2 cm skin wound was visible on the distal part of the right foot, with local redness and swelling at the wound edge, partial removal of liquefied necrotic tissue could be seen in the appearance, and exposed bone could be seen in the wound. The skin blood flow of the right foot was good, the sensation was reduced, and the active flexion and extension activities of each toe of the right foot were limited. The admission diagnosis was: infection after right foot injury and necrosis of some tissues after right foot injury. 10.7 Linezolid (0.6 g, ivgtt, q12h) anti-infection treatment was given, while wound secretion culture + drug sensitivity test was taken. 10.11 Wound secretion culture results: methicillin-resistant *S aureus*. 10.13 Debridement + necrotic tissue removal + wound vacuum sealing drainage was performed. The anesthesia method was local nerve block anesthesia, and linezolid was continued after surgery. From 10.13 to 10.15, Ketorolac tromethamine Injection (30 mg, ivgtt, bid) was given for analgesia. 10.15 The patient had elevated blood pressure (172/103 mm Hg) with mild headache; considering that Ketorolac tromethamine Injection caused increased blood pressure, was stopped 10.15, and the blood pressure reached 182/107 mm Hg on 10.16, accompanied by headache, and Nifedipine Tablets (10 mg, po, once)was given immediately. After retesting, the blood pressure dropped to 159/95 mm Hg, 10.16 to 10.19 Nifedipine Tablet (10 mg, po, qd), during which the blood pressure fluctuation value was 170–145/110–91 mm Hg. Linezolid was discontinued on 10.19, and blood pressure was 160–148/109–97 mm Hg from 10.20 to 10.21. Amlodipine besylate Tablets (5 mg, po, qd) was given since 10.22. According to drug sensitivity, Clindamycin phosphate Injection (0.8 g, ivgtt, q8h) was used to continue anti-infection treatment. From 11.10 to 11.18, combined with oral compound Sulfamethoxazole tablets (0.8 g, po, qd), the patient developed multiple body rashes with prurchiness, which was diagnosed as drug rash by dermatology. Stop compound Sulfamethoxazole tablets and Clindamycin phosphate Injection. 10.22 to 10.25 The patient was again given Ketorolac tromethamine Injection (30 mg, ivgtt, bid) for analgesia due to pain; 11.3, 11.5 Temporary administration of Tramadol extended-release tablet (0.1 g, po, once) for mild pain. From 10.22 to 11.15, the blood pressure of the patient fluctuated between 133–121/91–71 mm Hg; Amlodipine besylate Tablets were discontinued on 11.16; no obvious abnormality was found in the blood pressure of the patient from 11.16 to discharge. 12.19 Patients infected with coronavirus disease 2019 with fever, should be given Ibuprofen Tablets (0.2 g, po, tid) and recover after 2 days, showing no obvious effect on the underlying disease and blood pressure. 12.25 The patient’s rash completely resolved. 12.31 The wound healed well and the patient was discharged. No abnormal blood pressure was found in the follow-up of nearly 2 months after discharge.

## 3. Discussion

Hypertensive urgency are a type of Hypertensive crisis, has been described having an elevated blood pressure with systolic a blood pressure of greater than 180 mm Hg and a diastolic blood pressure of greater than 120 mm Hg without signs of end-organ damage. Patients can present with symptoms of headache, anxiety, pain, and upset stomach, which may lead to worsened hypertension and not be the cause.^[4]^ On the second admission, the patient developed Hypertensive urgency, which did not cause significant organ damage. The patient’s blood pressure increased at the second admission, and Ketorolac tromethamine Injection was discontinued first, but the blood pressure continued to rise. Nifedipine Tablets did not show a significant decrease in blood pressure, and Linezolid was then discontinued. On the second day after Linezolid was discontinued, the patient’s blood pressure began to decrease significantly, and Amlodipine besylate Tablets were given at the same time. There was no increase in blood pressure when Ketorolac tromethamine and Opioids were used again for pain. The patient’s blood pressure returned to normal on the 26th day after stopping Linezolid, and no abnormal blood pressure was found in the follow-up 2 months after discharge. The mechanism underlying this effect is unknown but in this case the strict temporal concurrence of hypertensive urgency and linezolid therapy seems to provide strong evidence for a causal relationship.

Hypertension caused by linezolid was rarely reported, and hypertensive emergency or hypertensive crisis caused by no obvious drug interaction was even rarer. Linezolid is a reversible monoamine oxidase (MAO) inhibitor, MAO inhibitor (MAOI), tricyclic antidepressants, selective serotonin reuptake inhibitors, analgesics, when these drugs are used in combination, they can cause symptoms such as serotonin syndrome, including high blood pressure. Serotonin syndrome presents as a range of excessive serotonergic effects that typically, severe hypertension is one of the clinical manifestations. Gunn et al,^[5]^ reported a case of hypertensive crisis after therapy linezolid with a MAOI. It has also been reported that linezolid combined with bupropion caused severe intermittent hypertension, high blood pressure as high as 260/145 mm Hg.^[6]^ However, our patient did not receive therapy with MAOI, bupropion and other related drugs, or a diet including large amounts of tyramine rich food. The surgical anesthesia method of the patient is foot nerve block anesthesia, and the nerve block anesthesia drugs usually contain opioids. Compared with general anesthesia, foot nerve block anesthesia does not require the use of general anesthesia drugs, so the possibility of systemic absorption is low. Relevant literature studies have shown that there may be systemic absorption of ropivacaine during nerve block anesthesia for knee replacement, but it is highly age-related, and appropriate dose reduction may be required for elderly patients.^[7]^ No hypertensive events occurred when the patient was first hospitalized with foot nerve block anesthesia, and there was no significant association between the occurrence of hypertensive emergencies and local opioid use. Therefore, we believe that the hypertensive emergency in patients is related to the use of linezolid, and there are no obvious factors that cause blood pressure increase when combined with other drugs. Studies have reported that linezolid can reversibly increase the pressurization effect of phenylpropanolamine or pseudoephedrine when given to healthy subjects with normal blood pressure. It can have an impact on blood pressure and heart rate.^[8]^

In this case, the patient’s blood pressure increased significantly on the 8th day of using linezolid again, blood pressure recovery time is about 1 month. It has also been reported that the blood pressure increased within 2 days of using linezolid. Within 48 h of discontinuation of linezolid, the blood pressure returned to normal.^[9]^ So the time of the adverse drug reactions and fade without a specific time limit.

To conclude, this case reminds the clinical use of linezolid, even in the absence of obvious drug interaction, linezolid can cause blood pressure increase, even hypertensive urgency. It is hoped that this case will remind clinicians that in the process of using linezolid, in the process of using linezolid, if there is a significant increase in blood pressure, the possibility of linezolid should be considered, and timely discontinuation or symptomatic treatment should be given to avoid serious clinical accidents.

## Author contributions

**Conceptualization:** Yiming Liu.

**Investigation:** Xinan Wu.

**Methodology:** Jingjing Luo.

**Writing – original draft:** Jingjing Luo.

**Writing – review & editing:** Xinan Wu.
